# Foie Gras Production: A Literature Review on the Welfare Implications of Force-Feeding of Ducks

**DOI:** 10.3390/ani16182838

**Published:** 2026-09-09

**Authors:** Bert Driessen, Lore Pellens, Béke Nivelle, Kirsten Van Rossem, Johan Buyse

**Affiliations:** 1 Laboratory of Morphology, Department of Morphology, Medical Imaging, Orthopaedics, Physiotherapy and Nutrition, Faculty of Veterinary Medicine, Ghent University, 9820 Merelbeke, Belgium; bert.driessen@ugent.be; 2 Animal Welfare Solutions, 3583 Paal, Belgium; lore@animalwelfaresolutions.com (L.P.); kirsten@animalwelfaresolutions.com (K.V.R.); 3 Nukamel, 6001 SE Weert, The Netherlands; 4 Laboratory of Livestock Physiology, Department of Biosystems, KU Leuven, 3001 Heverlee, Belgium

**Keywords:** foie gras, force-feeding, duck welfare, hepatic steatosis, animal welfare

## Abstract

Foie gras production is based on the force-feeding (gavage) of waterfowl to induce hepatic steatosis and subsequent liver enlargement. This practice has been widely debated due to concerns regarding animal welfare. This review provides a comprehensive overview of foie gras production, with a primary focus on the welfare implications of force-feeding in ducks, while contextualising historical and alternative practices involving geese. Evidence was evaluated across behavioural, physiological, pathological and health-related outcomes, together with procedural, housing and environmental factors. Endocrine findings are mixed, but repeated restraint and tube insertion can cause injuries to the upper digestive tract, while progressive feed administration produces marked liver enlargement, altered hepatic function and increased metabolic heat load. Reduced activity, locomotor impairment, lesions and elevated mortality have also been reported, although study designs and production methods and conditions vary. Alternatives under investigation include spontaneous fattening, microbiome-based interventions, cultivated-cell products and plant-based or processed analogues. Evidence concerning their welfare benefits, product quality, scalability and economic feasibility remains limited. Overall, the accumulated scientific evidence raises significant concerns regarding the welfare of force-fed birds and underscores the need for continued exploration of viable alternative production strategies.

## Simple Summary

Foie gras production relies on force-feeding (gavage) to induce hepatic steatosis and subsequent liver enlargement in waterfowl. While this practice is central to the industry, it has raised persistent animal welfare concerns. In recent years, public and regulatory scrutiny regarding the ethical implications of intensive animal farming systems has increased. This review synthesises current scientific knowledge on foie gras production, focusing specifically on the behavioural, physiological, and health-related welfare implications of force-feeding in ducks and geese. Evidence concerning physiological stress responses is inconsistent, with some studies reporting no increase in plasma corticosterone levels and other evidence indicating adverse behavioural, pathological or health-related outcomes. Reported injuries include oesophageal lesions, footpad dermatitis and carcass injuries. Force-feeding also produces marked liver enlargement due to fat accumulation, accompanied by changes in liver function. Scientific disagreement concerns whether these changes cause pain or suffering, the severity of the welfare compromise and the validity and interpretation of the indicators used. While alternative production methods that bypass force-feeding are being explored, they remain limited in scale and technological feasibility. This review highlights key research gaps, emphasises the need for objective welfare assessment frameworks, and aims to contribute an evidence-based perspective to the ongoing dialogue surrounding ethical animal production.

## 1. Introduction

The production of foie gras has become a contentious subject within the domains of animal welfare and ethical livestock production. Within the broader debate on the welfare and ethics of intensive animal production, the use of animals for food has come under growing societal scrutiny [1]. Foie gras production is particularly controversial due to the use of force-feeding (gavage), a practice intended to induce hepatic steatosis and substantial liver enlargement in ducks or geese [2].

The primary focus of public concern is the potential welfare implications of force-feeding, including its effects on animal behaviour, physiology, and health [3,4]. Concurrently, foie gras production remains embedded in long-established cultural, economic, and gastronomic traditions, particularly in Europe. This has resulted in a polarised debate involving differing ethical, scientific, and production-related perspectives.

This review synthesises the available scientific knowledge on foie gras production, with particular emphasis on the welfare implications of force-feeding in ducks. Evidence from geese is included where it provides relevant comparative, physiological, historical, or technological information. In addition to examining the effects of force-feeding, the review considers how current knowledge from animal behaviour, veterinary medicine, physiology, nutrition, pathology, housing, environmental management, and production technology can contribute to a more comprehensive welfare assessment and help identify opportunities to reduce specific adverse effects. Finally, the review evaluates current knowledge gaps and briefly considers production approaches that could avoid the need for force-feeding. This narrative review addresses three main questions: (1) which behavioural, physiological, pathological and health-related changes occur during force-feeding and how strong is the evidence that they compromise welfare; (2) which procedural, housing and environmental factors modify these outcomes; and (3) which alternatives to force-feeding have been investigated and what is known about their welfare implications and their technical and economic feasibility?

Industry organisations emphasise operator training, adapted equipment and group housing, and argue that waterfowl possess physiological features that permit high feed intake. Some of these features, such as a distensible oesophagus and the capacity for seasonal fat deposition, are biologically documented. They do not, however, demonstrate that commercial force-feeding is free from adverse welfare effects. These industry positions are therefore considered alongside evidence that production-induced hepatic steatosis exceeds natural seasonal fat deposition. The aim is to provide a balanced and evidence-based synthesis without advocating a predetermined ethical position.

## 2. Materials and Methods

### 2.1. Literature Search

A structured narrative literature review was conducted using Web of Science Core Collection, Scopus, and PubMed. The database search was conducted up to 30 July 2026. A final targeted update was performed on 3 August 2026 before preparation of the revised manuscript.

The first search focused on the welfare implications of force-feeding and used the following search string: (“foie gras” OR “force-feeding” OR “force feeding” OR gavage OR overfeeding OR “over-feeding”) AND (duck* OR goose OR geese OR waterfowl OR palmiped*) AND (welfare OR behaviour OR behavior OR stress OR pain OR fear OR avoidance OR health OR injury OR injuries OR lesion* OR mortality OR lameness OR locomotion OR “hepatic steatosis” OR hepatomegaly OR metabolism OR thermoregulation). The welfare search also included targeted combinations with corticosterone, oesophagitis, behaviour/behavior, mortality, lesion*, injury, thermoregulation and lameness.

A second search focused on alternatives to force-feeding and used the following search string: (“foie gras”) AND (duck* OR goose OR geese OR waterfowl OR palmiped*) AND (alternative* OR “spontaneous fattening” OR “voluntary overfeeding” OR microbiome OR probiotic* OR cultivated OR “cellular agriculture” OR “plant-based” OR “plant based”).

Terms in the alternatives search were selected a priori to cover four distinct approaches identified during scoping: spontaneous or voluntary fattening, microbiome or probiotic interventions, cultivated or cellular products and plant-based analogues.

The reference lists of relevant publications and major scientific reports were also screened. Targeted searches of governmental, institutional, and legal sources were conducted to identify scientific opinions, legislation, technical reports, and information on emerging production methods that was not available in peer-reviewed publications. In total, 94 sources were included and cited in the narrative review.

### 2.2. Eligibility and Study Selection

Publications were considered eligible when they addressed force-feeding in ducks or geese and reported behavioural, physiological, metabolic, pathological, health-related, or mortality outcomes. Studies examining the force-feeding procedure, hepatic steatosis, housing and management factors, or alternatives to force-feeding were also considered. Peer-reviewed original articles and reviews were included, together with relevant scientific opinions, conference proceedings, dissertations, technical reports, and legal documents.

Studies were classified as direct welfare evidence when they measured animal-based behavioural, physiological, pathological, clinical or mortality outcomes. Studies reporting only production, biochemical or technological outcomes were used as contextual evidence and were not treated as direct demonstrations of welfare effects.

Publications were excluded when they did not concern ducks, geese, or foie gras production. Sources were also excluded if they provided no information relevant to force-feeding, animal welfare, or alternative production methods, duplicated findings reported elsewhere, or contained insufficient information to support the narrative synthesis. Publications written in English, French, German, or Dutch were considered.

Titles and abstracts were screened for relevance to force-feeding, foie gras production, animal welfare, or alternative production methods. Potentially relevant publications were subsequently assessed in full text using the eligibility criteria described above. Duplicate records and sources that did not provide relevant information were excluded. Additional sources identified through reference-list screening and targeted searches were assessed using the same eligibility criteria.

### 2.3. Evidence Extraction and Synthesis

Information was extracted on the species or hybrid, study design, sample size, force-feeding procedure, housing and management conditions, welfare-related outcome measures, and principal findings. Outcomes were grouped into behavioural, physiological, metabolic, pathological, health-related, and mortality domains. Evidence concerning alternative production methods was grouped according to the production approach involved.

Because the included publications differed substantially in study design, force-feeding protocol, and outcome measures, the evidence was synthesised narratively and no meta-analysis was performed. Meta-analysis was not considered appropriate because no sufficiently large group of studies used comparable animals, force-feeding protocols, control groups, outcome definitions and measurement times. Greater weight was assigned to controlled peer-reviewed studies and authoritative scientific assessments than to descriptive sources or sources that are not peer-reviewed. Non-peer-reviewed sources were used primarily for legislation, historical information, commercial developments, and emerging technologies for which peer-reviewed evidence was limited or unavailable. Conflicting findings were retained and discussed.

No formal risk-of-bias tool was applied to each individual study, as this was a narrative review. Nevertheless, study design, sample size, the presence of an appropriate comparator, clarity of the force-feeding procedure, validity of the welfare indicators, and consistency across outcome domains were considered qualitatively when interpreting the evidence. Studies with small sample sizes, no appropriate comparator, incompletely described force-feeding procedures, or welfare indicators of uncertain validity were interpreted cautiously and were not considered sufficient on their own to establish causal welfare effects.

The restriction to publications written in English, French, German, or Dutch may have excluded relevant evidence published in other languages and therefore creates a risk of language bias. Because this narrative review included heterogeneous experimental, observational, historical, legal, and technical sources, no single risk-of-bias instrument was considered applicable across the entire evidence base. Study quality was therefore assessed qualitatively rather than by applying different formal instruments to individual source categories. Consequently, the conclusions should be interpreted in light of possible selection and reporting bias, as well as confounding caused by differences in study design, housing, management and production stage.

## 3. Foie Gras Production

### 3.1. Foie Gras

The French term ’foie gras’ can be translated as ‘fatty liver’, referring to the enlarged, fat-rich liver of a duck or a goose [5]. A more accurate description would be ‘fattened liver’, since the liver enlargement is induced through force-feeding rather than voluntary hyperphagia. The animals are fed a diet that is high in energy via a tube inserted into the oesophagus [6]. This results in hepatic steatosis, marked by a substantial increase in both liver size and fat content, with the liver becoming several times larger than under normal physiological conditions [3,7,8].

SCAHAW [7] described several commercial categories of foie gras products that differ in their physical presentation and proportion of fatty liver. Foie gras entier consists of whole fatty liver presented in one piece, whereas foie gras consists of portions of fatty liver and bloc de foie gras is a reconstituted preparation in which the original liver pieces are no longer distinguishable. Preparations containing lower proportions of fatty liver include parfait de foie (at least 75%), médaillon de foie, pâté de foie, galantine de foie and mousse de foie (approximately or at least 50%, depending on the product category), and produits au foie gras (at least 20%) [7]. These categories describe the composition and presentation of commercial products rather than animal-welfare outcomes. They are nevertheless included to clarify the different products marketed under foie gras-related designations and the commercial context in which fatty liver is produced.

Until March 2026, Commission Regulation (EC) No 543/2008 defined foie gras for EU poultrymeat marketing purposes and prescribed minimum liver weights of 300 g for ducks and 400 g for geese. That Regulation was repealed by Commission Delegated Regulation (EU) 2026/343, while detailed implementing rules are laid down in Commission Implementing Regulation (EU) 2026/344 [9,10,11]. The new regulatory framework does not reproduce the former minimum liver-weight requirements or an equivalent specific marketing definition of foie gras. The removal of these requirements eliminates a regulatory minimum-liver weight threshold, but there is yet no evidence demonstrating that it has changed commercial production targets, force-feeding practices or animal-welfare outcomes. In France, foie gras preparations remain subject to national product requirements, including a maximum melting rate of 30%, and foie gras is legally recognised as part of the country’s cultural and gastronomic heritage, with force-feeding identified as the production method [12,13,14,15]. The melting-rate criterion is a product-quality requirement and should not be interpreted as an animal-welfare threshold.

### 3.2. History

Force-feeding of waterfowl has a long history, with evidence of the practice extending from ancient Egypt and the Mediterranean world to its later development in Europe [16,17,18]. Historical accounts associate the discovery and appreciation of fattened waterfowl with the seasonal accumulation of energy reserves, including body fat and enlarged livers [16]. Until the mid-twentieth century, foie gras production remained limited in volume and relied predominantly on geese, whose seasonal breeding pattern restricted year-round production [8,16]. Over time, foie gras production became more specialised and commercially organised, while feeding methods, housing systems, equipment and production objectives changed. During the twentieth century, production shifted predominantly from geese to mulard ducks, a sterile hybrid between the Muscovy duck (*Cairina moschata*) and the domestic duck (*Anas platyrhynchos*), owing partly to production efficiency, growth characteristics and suitability for fatty-liver production [8].

This historical development is relevant to animal-welfare assessment because the species used, production scale, housing conditions, feeding procedures and technical execution of force-feeding have changed over time [7,8,16,17,18]. Consequently, historical descriptions of foie gras production cannot automatically be extrapolated to contemporary commercial systems, while findings obtained under current production conditions may not fully represent earlier practices. Moreover, the long cultural history of foie gras does not in itself provide evidence that contemporary production methods are acceptable from an animal-welfare perspective. The ethical, societal and scientific assessment of foie gras production has evolved alongside changing knowledge and expectations concerning animal health and welfare [3,4,6,7]. Historical context therefore helps distinguish the cultural continuity of foie gras production from the separate scientific question of how contemporary production affects the welfare of ducks and geese.

### 3.3. Genetics

The mulard duck is the cornerstone of modern professional foie gras farming. It is a sterile intergeneric hybrid produced by crossing the male Muscovy duck (*Cairina moschata*) with a female domestic duck (*Anas platyrhynchos*), predominantly the Pekin breed [4,19,20]. Although the Pekin duck is the most common maternal line used for this cross, Rouen ducks are occasionally employed as well [21]. This specific intercross results in a sterile hybrid due to distinct differences in chromosome size and number between the two parental species [22].

Sterility is an expected genetic consequence of the interspecific cross used to produce the commercial mulard hybrid [21,22]. It is a reproductive characteristic and should not, in itself, be interpreted as evidence of impaired welfare. From an animal-welfare perspective, potentially more relevant genetic effects include correlated responses to selection, genotype-specific susceptibility to disease or metabolic disorders, and functional limitations associated with commercially selected traits [21,23]. The literature identified in this review provided no direct evidence that the sterility of mulard ducks itself causes adverse welfare outcomes.

The production of foie gras is almost entirely reliant on the utilisation of male mulards. For instance, in France, 96% of the production is attributable to male mulards [24,25]. In the parental lines, the selection of better carcass quality and conformation is driven by the objective of developing a larger pectoral muscle, known as ’magret’ [26]. This large pectoral muscle plays a key role in the overall meat yield and value of the force-fed bird. The female specimens are primarily used for meat production, yet their application is expanding to include foie gras production. However, the foie gras of female birds is of a lower quality than that of male birds. For instance, the carcass and liver weights of males are approximately 10% higher [27]. In addition, the trial by Marie-Etancelin et al. [27] revealed that one-third of the livers of female ducks exhibited moderate to high levels of veining, while 99% of the livers of males demonstrated low levels of veining. Furthermore, male mulards generally produced foie gras with lower melting rates and less pronounced veining, resulting in a superior technological quality compared with females. It has been demonstrated that wild species of domestic ducks, such as the mallard, sometimes migrate according to season and weather conditions [28]. The Muscovy duck inhabits subtropical to tropical climates in Central and South America and is not a migratory species [29]. Migratory birds store feed reserves just before migration (‘pre-migratory hyperphagia’ and subsequent nutrient/energy storage). The liver is one of the organs responsible for storing reserve nutrients. Although seasonal fat deposition occurs in migratory waterfowl, the degree of hepatic steatosis induced during foie gras production greatly exceeds the level of physiological seasonal fat accumulation [7,26].

### 3.4. Morphology and Histology

Ducks and geese lack a well-defined crop, in contrast to the majority of other bird species, such as *Gallus gallus domesticus* [26,30]. Instead, a spindle-shaped dilatation of the oesophagus, often termed ‘pseudo-crop’, is observed. The repeated filling of the oesophagus has been shown to increase its capacity [4,26]. In the wild, oesophageal dilation enables these avian species to accommodate and store considerable quantities of feed for later digestion [31]. Guy et al. [32] reported that a single male mulard can ingest up to 500 g of feed in a single meal and over 750 g of feed during a day, without any physical constraint. The SCAHAW [7] stated in 1998 that ducks and geese possess protective reflexes that may function similarly to the gag reflex when fluids enter the trachea. In the context of force-feeding birds, this reflex must be overcome, a process which can initially result in stress and injury. However, there is no consensus regarding the presence or absence of a gag reflex [26]. Producers of foie gras posit that if force-feeding is executed correctly, the force-feeding tube will not result in the occlusion of the upper respiratory tract, given that the trachea and oesophagus are fully separated [33]. It is not evident that birds experience discomfort or regurgitation [34]. Mulards are characterised by a combination of anatomical and behavioural traits that are derived from both species. For instance, the birds possess claws reminiscent of Muscovies, yet, as domestic ducks, they rarely engage in arboreal activities [35]. In the context of flight reactions, the potential for injury through claw-on-claw contact is a distinct possibility.

### 3.5. Production and Consumption of Foie Gras

The European federation of foie gras producers, Euro Foie Gras [36], is a prominent organisation in the field of foie gras production. The organisation was established in 2008 and comprises members from the five foie gras-producing countries in Europe: Belgium, Bulgaria, France, Hungary and Spain. In recent years, approximately 21,000 tonnes of foie gras have been produced annually in the EU, with the majority originating from duck liver (approximately 95%), and a smaller share from goose (approximately 5%) [36]. Approximately 80% of the global foie gras production is sourced from Europe. The other primary producers on a global scale are China, the USA and Canada [36]. The foie gras market is also present in major non-European markets, including Japan, China, the United States, Canada and Israel. Among these, Japan is regarded as one of the most important export destinations for French foie gras products [24]. In several (European) countries, the practice of force-feeding is prohibited by law. However, these countries also import foie gras [26]. A prohibition on domestic force-feeding combined with continued importation limits the direct geographical reach of national animal-welfare legislation, because the production practices associated with imported foie gras occur in another jurisdiction. This situation may displace production towards countries where force-feeding remains permitted rather than eliminate the production practices targeted by the national prohibition. France continues to dominate the foie gras industry, ranking first in production, consumption, and exportation on a global scale. Duck liver constitutes over 95% of French foie gras production, with goose foie gras representing a comparatively negligible percentage of the total output. Recent years have seen considerable fluctuations in French production, largely attributable to the recurrent emergence of highly pathogenic avian influenza. The decline in production, from approximately 18,000–19,000 tonnes prior to the major avian influenza outbreaks to around 9000–10,000 tonnes in 2022, was followed by a subsequent recovery following the implementation of a nationwide duck vaccination program in 2023. Production reached approximately 15,800 tonnes in 2024 and exceeded 16,800 tonnes in 2025 [24]. On average, foie gras consumption in France remains among the highest globally, at around 280 g per capita per year [36]. Belgium is the second largest consumer and importer of raw foie gras worldwide. Per capita consumption remains relatively high in comparison to other countries (approximately 105 g per capita per year), whilst domestic production is restricted and primarily focused on ducks, accounting for only a minor proportion (approximately 13 tonnes in 2024) of the total EU production [36].

### 3.6. Feed

Maize is the most prevalent cereal in the diets of foie gras-producing birds, particularly during the force-feeding period [8,25]. The high starch content encourages the accumulation of liver fat [37]. In conventional foie gras farms, ducks and geese are subjected to a dietary regime consisting of cooked, salted whole maize grains (70% maize and 30% water). A small amount of fat (less than 2%) is incorporated into the mixture to facilitate the movement of the grains through the oesophagus during the force-feeding process. In modern foie gras farms, the birds are typically force-fed a mixture of uncooked maize and water. In foie gras production systems, maize is commonly administered during the force-feeding period. This is typically either as maize flour or as a mixture of whole grains and maize flour [8,25,38]. The utilisation of diets comprising maize flour is predominantly favored by producers due to the ease and expediency with which they can be administered. However, they may result in lower liver weights and reduced foie gras quality compared with diets containing a higher proportion of whole maize grains [25,38]. Lower liver weight is primarily reported in these studies as a production and product-quality outcome and should not automatically be interpreted as evidence of improved welfare. Liver weight and corticosterone should be assessed separately: a reduction in proportional liver weight without a corresponding change in plasma corticosterone would not by itself demonstrate a welfare benefit. Although a lower final liver weight could theoretically reduce some risks associated with pronounced hepatomegaly, any welfare benefit would need to be demonstrated using animal-based behavioural, physiological, pathological and clinical indicators [7,26].

### 3.7. Sensory Characteristics

Sensory and technological characteristics are relevant to this review because substantial differences in texture, taste, appearance or processing properties may affect consumer acceptance and the commercial adoption or introduction of alternatives to force-feeding [1,39]. These product characteristics are not animal-welfare indicators, but they may influence the technical feasibility and potential uptake of production systems that avoid force-feeding [1,39].

Evidence concerning the sensory properties of foie gras produced without force-feeding remains limited and is derived mainly from geese rather than from mulard ducks. Fernandez et al. [39] compared livers from conventionally force-fed geese with those from geese fattened spontaneously through a programme simulating autumn conditions. The conventionally produced livers received higher overall scores from trained assessors and differed from the alternative livers in colour, texture, odour and bitterness. These differences were partly associated with the lower fat content and different pigment accumulation of the alternatively produced livers [39]. However, the extent to which these findings can be extrapolated to mulard ducks is uncertain because genotype and hepatic lipid metabolism influence product characteristics [21,37,39]. Further research should therefore assess whether alternatives can achieve acceptable product characteristics while evaluating their animal-welfare effects separately using appropriate animal-based indicators.

### 3.8. Management

Producers have developed a specific feeding schedule for ducks for the pre-fattening period. The utilisation of this scheme results in a reduced length of the force-feeding period when compared with its length in geese [40,41]. Force-feeding programmes are species-specific. In commercial production, mule ducks are generally force-fed twice daily for approximately 10–15 days, whereas geese are commonly force-fed for a longer period and may receive more meals per day. The exact duration and feeding frequency vary according to genotype, management system and the targeted liver characteristics [7,11,40]. The feeding scheme for ducks is to be divided into four stages [7,8]. The final stage, referred to by the European Food Safety Authority (EFSA) as the overfeeding period, is recognised as a distinct production phase in foie gras production systems [23]:

- Starting period: ducks are fed a concentrate ad libitum between hatching and the 6th to 9th week of life. They are mainly housed in stables on straw, with free-range if the weather allows.
- Growing period: during 3 to 5 weeks, the concentrate intake is restricted, either by reducing the amount of concentrate or by limiting the period in which it is available. Ducks have outside access during the day, where they also can graze on grass.
- Pre-force-feeding period: ducks are provided with ad libitum access to concentrate over a period of 3 to 10 days. The quantity of feed available per bird is augmented on a daily basis. Firstly, the aim is to enlarge the oesophagus, or, more specifically the oesophageal outpouching, also known as the pseudo-crop. Secondly, the digestive tract and its secretions are stimulated, thus preparing it for the processing of large quantities of feed. Furthermore, the process of liver fattening has already been initiated. Consequently, the weight of the duck liver can increase to approximately 180 g at the end of this period, in contrast to the typical range of 80 g under normal circumstances. During the day, the ducks have outside access, where they can also graze on grass.
- Force-feeding period: approximately 12 to 14 weeks post-hatching, the ducks are transferred to indoor housing, comprising group cages or small pens, where the force-feeding process starts. For a period of 10 to 15 days, a portion of an energy-rich feed is administered twice a day through a tube that is manually inserted into the oesophagus [14]. A force-feeding period of 10–15 days with two feeding events per day exposes each bird to approximately 20–30 handling and tube-insertion events. The cumulative welfare relevance therefore depends not only on the response to an individual feeding event but also on the repeated effects of restraint, tube insertion, feed administration and recovery [7,26,42]. Management and human–animal interactions during the preparatory period may also influence digestive capacity, fear of humans and subsequent behavioural responses to force-feeding [7,8,43,44]. Published and commercial sources describe the use of group cages or small pens, but their dimensions, group sizes and usable floor areas vary among systems and were not consistently reported in the literature reviewed. Consequently, no single “standard” cage or pen dimension is presented here. Future studies should report these housing variables explicitly because space allowance and group size may affect mobility, simultaneous resting and lesion risk [23,26,45]. The duration and execution of this final force-feeding stage directly dictate the subsequent technological yield and market classification of the liver. The feed is pushed through the tube into the birds’ pseudo-crop [4]. The feed is characterised by a high carbohydrate content, a low protein content, and an imbalanced amino acid composition [46]. In general, the feed is composed primarily of maize, which is boiled and mixed to ease digestion [7]. Initially, each duck is provided with a quantity of 180 to 200 g of broken maize. The portion is then increased in a gradual manner to 450 g per meal at the end of the force-feeding period. During this period, the ducks are accommodated in small groups within cages in a room that is temperature and relative humidity-controlled room [26,40,47]. The ducks are typically accommodated on a steel slatted floor (galvanised or stainless) or, to a lesser extent, on a plastic slatted floor [4]. This process of fattening is applied throughout the year in an air-conditioned room.

### 3.9. Legislation

At the European level, the production of foie gras is subjected to regulation in accordance with Council Directive 98/58/EC, which pertains to the protection of animals kept for farming purposes [48]. Moreover, the Standing Committee of the European Convention for the Protection of Animals Kept for Farming Purposes adopted a series of recommendations concerning Muscovy ducks (*Cairina moschata*) and hybrids of Muscovy and domestic ducks (*Anas platyrhynchos*) [45]. These recommendations specify, among others, that flooring should be designed and maintained to prevent discomfort, distress or injury, that all birds should have sufficient space to rest simultaneously, and that appropriate bedding should be provided and maintained in a dry condition whenever possible. Furthermore, environmental conditions should allow the birds to maintain plumage condition and express species-specific behaviours.

Recent EFSA evaluations have concluded that several extant European recommendations for the welfare of ducks and geese are outdated and do not fully reflect current scientific knowledge, thereby highlighting the necessity for updated welfare standards [23]. Commission Regulation (EC) No 543/2008 [11], which remained in force until March 2026, defined foie gras as a liver from ducks or geese that had been “fed in such a way as to produce hepatic fatty cellular hypertrophy”. The regulation further required duck foie gras to be completely bled, of uniform colour and to weigh at least 300 g.

French legislation (article L654-27-1) [15], protects foie gras as both a cultural and gastronomic heritage, and even designates ’gavage’ (force-feeding) as the production method for foie gras. This statutory recognition may contribute to the political and cultural complexity of reform, but it does not itself provide scientific evidence concerning animal welfare. Cultural recognition and animal-welfare assessment therefore constitute distinct, although interacting, legal and policy considerations [15,48]. Since January 2016, the individual housing of ducks in cages for foie gras production has been prohibited in France, and birds must be housed in group systems.

The legislative framework pertaining to the welfare of animals with regard to foie gras production varies considerably between European countries and regions. Several countries have prohibited force-feeding, either directly or indirectly through general animal welfare legislation. In Belgium, important legislative changes have occurred in recent years. Following the adoption of legislation by the Flemish Parliament in 2019, force-feeding for foie gras production was phased out and became prohibited in Flanders in 2023. Consequently, foie gras production by force-feeding has ceased in Flanders. Conversely, foie gras production remains permitted in the Walloon Region under the applicable animal welfare legislation [49]. The Flemish prohibition concerns the production of foie gras by force-feeding and does not constitute a general prohibition on the importation, sale or consumption of foie gras lawfully produced elsewhere. Consequently, foie gras remains commercially available in Flanders despite the prohibition on domestic production by force-feeding [49].

Consequently, foie gras production within Europe is increasingly characterised by differing national and regional legal frameworks, reflecting ongoing societal, ethical and political debate regarding the compatibility of force-feeding practices with contemporary animal welfare standards. The increasing divergence in legislation between countries is indicative of the ongoing controversy regarding the welfare implications of force-feeding practices, and highlights the lack of consensus at the European level.

## 4. Welfare Aspects

While these production practices are technically efficient, they raise critical questions regarding animal welfare, which are discussed in the following section.

### 4.1. Force-Feeding

At the end of the force-feeding period, each of the two daily feed portions may reach 400–450 g [7,14,46]. Comparisons with voluntary feed intake should be interpreted cautiously because intake varies with age, production stage, prior feed restriction, diet composition and measurement conditions. Nevertheless, repeated administration of such quantities represents a marked deviation from normal voluntary feeding patterns. Ducks and geese possess a distensible oesophagus that enables them to ingest relatively large feed items and amounts [7,30,31,46]. However, anatomical, behavioural and physiological differences between species mean that evidence obtained in geese should not automatically be extrapolated to mulard ducks, or vice versa [7,23,26]. Force-feeding involves the insertion of a feeding tube into the oesophagus. Rochlitz and Broom [26] describe this tube as rigid, whereas tube characteristics are not consistently reported in the other reviewed sources [7,46]. Consequently, the available evidence does not allow the welfare effects of tube design to be distinguished from those of equipment condition, insertion technique, operator skill and other procedural factors [7,26,42]. The repeated insertion of the feeding tube (also known as the feed probe) may cause injuries to the beak and oral cavity, as well as abrasion, inflammation and focal lesions of the pharyngeal and oesophageal mucosa. The risk and severity of such lesions depend partly on operator skill, equipment condition and the speed and accuracy of tube insertion [7,26,42]. Therefore, Euro Foie Gras [36] stipulates in its charter that force-feeding should only be carried out by personnel with the relevant skills and experience, and that the equipment used must be adapted well-maintained, with the aim of minimising the risk of injury to the birds.

Birds possess a multitude of pain receptors and an extensive pain recognition system. The beak and throat area, also called the oropharyngeal area, is particularly sensitive. The introduction of a feed tube for the purpose of force-feeding can result in tissue damage, which, in turn, can cause pain [7].

The gagging or pharyngeal reflex is defined as a reflex contraction of the muscles located at the posterior region of the throat, initiated by the contact of the palate, the back of the tongue, the area surrounding the tonsils, or the posterior region of the throat. The gagging reflex is a protective mechanism that prevents the entry of feed particles into the trachea and the subsequent entry of objects into the oesophagus, with the exception of those that are naturally part of the swallowing movement. Furthermore, the reflex serves as a protective mechanism against suffocation [7,26].

The question of whether mulards possess a gag reflex remains a subject of debate, with divergent views being expressed on the matter [3]. The SCAHAW [7], Duncan et al. [20] and Broom and Rochlitz [26] posit that ducks possess a gag reflex, citing characteristics of their feeding behaviour as supporting evidence. It is posited that, in contrast to the behaviour of pelicans and storks, which have been observed to consume substantial pieces of feed with ease, mulards do not engage in such extensive ingestion. Birds are devoid of teeth and are anatomically adapted to swallow food whole [50]. In the context of waterfowl, the oesophagus functions as an elastic, muscular tube that possesses the capacity to expand, thereby accommodating substantial food items [40]. This property naturally facilitates the passage of intact, unchewed grains. Klingler [50] investigated the tracheal and oesophageal displacement (TED) in birds. The manner of feeding was identified as a probable explanation for the occurrence of TED.

The welfare relevance of this debate is not that the presence of a gag or pharyngeal reflex would itself demonstrate pain. Such a reflex is a protective response to stimulation of the pharyngeal region [7,26]. If elicited or repeatedly overridden during tube insertion, it may indicate aversive stimulation and could increase struggling and the risk of tissue injury. Conversely, the absence of this reflex would not demonstrate that tube insertion is painless, because pain or discomfort may arise through other mechanisms, including handling, pressure, stretching, abrasion or tissue damage in the oropharyngeal and oesophageal regions [7,26]. The available evidence therefore does not support using the presence or absence of a gag reflex as a stand-alone indicator of pain or welfare during force-feeding.

The process as a whole, and various constituent elements such as handling, tube insertion and forced administration of large quantities of feed, have the potential to impact the well-being of the birds. It has been demonstrated that the effects may accumulate as a consequence of the repeated nature of the procedure, which is generally performed twice daily for approximately 14 consecutive days [26].

The extent to which force-feeding induces a physiological stress response remains a subject of debate. Guémené et al. [51] found no significant increases in plasma corticosterone concentrations, no changes in adrenal responsiveness to ACTH stimulation, and no significant changes in heterophil-to-lymphocyte ratios during the force-feeding period. On this basis, the authors concluded that, under the experimental conditions employed, force-feeding did not appear to be perceived as an acute or chronic stressor by male mulards. However, corticosterone represents only one component of the physiological stress response. A reduced endocrine response during a repeated procedure could reflect habituation or altered responsiveness of the hypothalamic–pituitary–adrenal axis, but these explanations were not tested directly in the cited force-feeding study [50]. Regardless of the underlying explanation, a limited or blunted corticosterone response does not exclude pain, fear, tissue injury or behavioural restriction [7,26]. Welfare conclusions should therefore integrate endocrine findings with behavioural observations, clinical examination, pathological findings, mobility, morbidity and mortality rather than relying on plasma corticosterone levels alone [7,26,38]. Outcomes may also depend on operator skill, equipment condition and insertion technique [7,26,48]. Consequently, results obtained under controlled conditions with experienced personnel cannot automatically be extrapolated to every farm, flock or production batch.

The process of tube feeding has been associated with the occurrence of pain and tissue damage, particularly when the procedure is conducted in an expeditious manner or is not performed in accordance with the established protocols. Throughout the process of feeding, a rigid tube is repeatedly inserted into the upper digestive tract. Consequently, the condition of the oesophagus before, during and after the force-feeding period is of particular importance. Several studies have investigated histological and neurophysiological indicators of tissue damage and pain in ducks during force-feeding. Servière et al. [42] described moderate, subacute and multifocal oesophageal inflammation (oesophagitis) in force-fed birds. Local inflammatory lesions may develop in the upper digestive tract as a result of repeated insertion of the feeding tube and the passage of feed boluses, which can stretch and abrade the oesophageal wall. Servière et al. [42] also conducted a comparative study between force-fed ducks and ducks in which oesophageal necrosis had been experimentally induced using a mustard oil-based irritant under anaesthesia. The activation of both the peripheral and central neurons indicated strong pain signalling in the brains and spinal cords of the experimentally treated birds. In contrast, only limited activation was observed in force-fed ducks. These findings suggest that force-feeding may induce oesophageal irritation and inflammation, although to a lesser extent than experimentally induced oesophageal injury. The lower neuronal activation observed following commercial force-feeding may possibly reflect differences in the severity or duration of the oesophageal lesions compared with the experimentally induced injury [48]. However, lesion severity and duration were not independently manipulated or matched in that study. The difference in neuronal activation therefore cannot be attributed conclusively to either factor, nor can limited activation be interpreted as demonstrating the absence of pain or adverse experience during commercial force-feeding.

### 4.2. Liver

It has been observed that wild animals with an irregular diet (quantitative and qualitative) are often adapted to store nutrients when a large meal is consumed. Such a system may also be activated when ducks and geese absorb large quantities of feed [7]. The process of foie gras production, which involves the induction of liver fattening in many bird species, utilises a specific fat storage mechanism. This mechanism is called lipogenesis and takes place in the liver. Normally, lipids are transported from the liver to other parts of the body as lipoproteins of varying density via the blood [46]. However, an increase in feed intake can result in the accumulation of fat in the liver. The liver weight of ducks exhibits seasonal variations, with a potential increase ranging from 30 to 50% during certain periods. In the case of female ducks, these changes are more pronounced. More pronounced steatosis in some domestic genotypes reflects an imbalance in which hepatic *de novo* lipid synthesis and lipid uptake from the portal circulation exceed hepatic lipid export in circulating lipoproteins of different density and subsequent utilisation by peripheral tissues. This imbalance is accentuated by the high-carbohydrate diets used during force-feeding [36,43,52].

It has been proposed that domestic ducks tolerate liver enlargement as an adaptation related to the accumulation of reserves before winter migration [3]. However, the analogy between force-feeding-induced hepatic steatosis and natural migratory fattening is limited. Mulards are sterile production hybrids and do not undergo normal migration [21,25,28]. Although mallards and other migratory waterfowl may accumulate seasonal energy reserves before migration (premigratory fattening), the liver enlargement and hepatic lipid accumulation induced by force-feeding greatly exceed the seasonal changes reported in wild waterfowl [7,26]. Neither mulards nor their parental lines develop comparable liver enlargement when maintained under standard farming conditions without force-feeding [26]. Consequently, natural migratory fattening should not be used as evidence that the hepatic changes induced by force-feeding are physiologically normal.

In force-fed ducks, the hepatocytes and other cells in the liver undergo changes. The increase in the number of fat globules in the cells is arguably one of the most evident changes. In certain circumstances, there may be a limited increase in the number of fat globules in the liver of ducks, but not to the extent that occurs in birds after force-feeding. During the force-feeding period, significant alterations in liver physiology have been reported [7]. The question of whether this degree of steatosis should be considered pathological remains a matter of debate among pathologists. Hepatic steatosis can regress following the cessation of force-feeding. Earlier studies in geese and mulard ducks reported that liver weight and lipid content returned towards basal values within several weeks [7,53], while a recent study in male mulards found that most physiological and biochemical parameters, including liver mass and plasma liver-enzyme activities, returned to control values within approximately 20–29 days [54]. Reversibility demonstrates recovery potential but does not establish that the preceding condition was physiologically normal or free from adverse experience. This distinction is particularly relevant in light of the cellular stress, hypoxia, inflammatory responses and apoptotic changes reported during the force-feeding period [54,55,56]. However, Rémignon et al. [55] investigated the extent of apoptosis of liver cells in force-fed mulards by measuring the activity of caspase-3, -7, -8 and -9. The force-feeding process was conducted in accordance with the standard commercial protocols. No marked increase in caspase activity was measured until the eighth day of force-feeding. However, on days 10 and 12, a significant increase in the activity of all measured caspases was observed. These findings suggest the onset of hepatocyte apoptosis and a loss of cellular homeostasis in at least some force-fed ducks [55]. Recent experimental work has demonstrated that the development of hepatic steatosis in force-fed mulards is accompanied by oxidative stress, tissue hypoxia and inflammatory responses, particularly towards the end of the force-feeding period. This suggests that the process is not merely a simple accumulation of lipids but also involves cellular stress [56]. Force-feeding markedly increases liver weight and hepatic lipid deposition. Typical foie gras livers weigh approximately 300–600 g in ducks and up to 900 g in geese, although substantially higher liver weights have occasionally been reported in mulards. It has been established that the hepatic lipid content of force-fed ducks generally exceeds 50%, in comparison to approximately 70–80 g and less than 10% lipid in non-force-fed ducks [8,37,55]. Recent data from force-fed mulards demonstrated an increase in liver weight from approximately 125 g before force-feeding to 525–573 g at the end of the force-feeding period, accompanied by an increase in hepatic lipid content from about 5% to more than 58% [56]. An increase in liver size and fat content has been demonstrated to be associated with altered hepatic function, including reduced hepatic blood flow and delayed metabolic clearance of substances [57]. The diet of ducks consists of substantial amounts of maize, which is characterised by a high starch content and low fat and protein levels. This dietary composition is known to stimulate fat synthesis in ducks. The elevated starch content has been demonstrated to induce an escalation in insulin and glucose levels, which in turn have been shown to stimulate the transcription factors SREBP-1c and ChREBP. These transcription factors subsequently stimulate glycolysis and lipogenesis [28,58]. In order to ensure that ducks produce as little as possible Very Low Density Lipoprotein (VLDL), it is necessary to limit the nutrients that are required for the synthesis of VLDL, such as the amino acids methionine and choline in the diet [26,28]. In contrast, thiamine and biotin are abundantly present in maize. The presence of these constituents is indispensable for the conversion of sugar into fats [26]. Liver fattening is the result of a complex interplay between various processes within the liver, particularly the increased capacity of *de novo* lipogenesis in hepatocytes, inadequate export of triacylglycerols from the liver, and a limited capacity of peripheral tissues to take up circulating lipids. These processes result in the return of triglycerides to the hepatocytes, where they accumulate and trigger the development of liver steatosis. Not only the species but also the genotype has the capacity to contribute to the development of hepatic steatosis [52,59]. The reduced ability of the liver to detoxify at the end of the force-feeding period has been interpreted as indicative of altered hepatic function and potential clinical pathology [7]. The reduction in detoxification is demonstrated by the slower degradation and longer half-life of bromosulfophthalein (BSP) and indocyanine green (ICG), as well as by the delayed absorption of antibiotics compared with non-force-fed ducks at the end of the force-feeding period. Increased levels of liver-associated enzymes in the blood, resulting from damage to hepatocyte cell membranes, provide further evidence of altered liver function. These findings suggest that severe hepatic steatosis is associated with altered hepatic function. This is evidenced by a range of factors including morphometric, biochemical, histological and pharmacological aspects [7]. It remains unknown whether these hepatic alterations cause pain in force-fed ducks. Oxidative stress, tissue hypoxia and inflammatory responses provide evidence of cellular stress, while the increased caspase activity reported towards the end of force-feeding is consistent with the onset of apoptosis and loss of cellular homeostasis [55,56]. These findings do not directly measure pain. However, marked hepatomegaly and associated tissue changes could plausibly contribute to discomfort, including through compression of adjacent structures [7,26]. To our knowledge, the occurrence and severity of any resulting pain in force-fed ducks have not been quantified directly.

### 4.3. Behaviour

#### 4.3.1. Time Use

Force-fed ducks appear to allocate a greater proportion of their time to sitting than to standing. In the context of duck husbandry, the restriction of movement in slatted-floor housing, whether in small groups or individual cages, can impede the ability of ducks to perform essential behavioural functions [7,60]. This restriction can hinder activities such as free movement, wing flapping, preening, exploration, and social interaction, which are all integral to their well-being and health. The process of force-feeding entails the capture and restraint of the bird, followed by the rapid insertion of the feeding tube and subsequent distension of the oesophagus. These actions have the potential to engender aversion and discomfort, both during and immediately after feeding [4]. During the first week of the force-feeding period, mulards gradually allocate more time to rest. The question whether this trend will continue in the second week of force-feeding remains unanswered [7]. Behaviours such as grooming and smoothing of the feathers are observed less frequently and for shorter periods in force-fed birds [7,26]. At the end of the force-feeding period, a reduced frequency of tail wagging was observed in the ducks compared to the birds in the control group. Furthermore, the force-feeding of ducks resulted in reduced ambulatory behaviour and an increased propensity for recumbency. Conversely, the head was positioned in a less-rested state. According to SCAHAW [7], the ducks in the experimental group demonstrated a reduced flapping and head-shaking frequency during the force-feeding period. Conversely, Broom and Rochlitz [26] assert that the ducks in the experimental group demonstrated an increased flapping and head-shaking frequency during the force-feeding period. There was no evidence of aggressive behaviour towards conspecifics. However, the ducks exhibited increased agitation during the first two to three days of the force-feeding period [7,26]. Increased sitting or recumbency cannot be assumed to represent a freely chosen resting preference. It may reflect increased body and liver mass, reduced locomotor capacity, altered metabolic state, housing constraints, heat load or discomfort [7,26,61]. Faure et al. [43] reported avoidance-related responses towards the force-feeder, whereas Stomp et al. [61] found that behavioural reactions during force-feeding were influenced by housing conditions. These findings indicate that some components of handling, restraint or feeding may be aversive in at least some contexts. However, the reported responses do not establish that every bird is equally averse to the caretaker, the administered diet or the entire force-feeding procedure. Behavioural changes should therefore be interpreted in combination with the production stage, housing conditions and other behavioural, clinical and physiological indicators. According to Hoffmann [29], the behaviour of mulards is more similar to that of Muscovy ducks than to that of domestic ducks. However, it should be noted that mulards move more slowly and spend more time on water than either of these duck species. Concurrently, certain behavioural characteristics of the former resemble those of domestic ducks. Mulards have been shown to be generally unable to fly [29]. Wild Muscovies and mulards spend a large part of their active time searching for and investigating potential sources of sustenance, thereby engaging in effective foraging [7]. The wild Muscovy duck forages both on the water and on dry land, where it may graze, dig or hunt [62]. Feather pecking, which occasionally results in instances of cannibalism, has been identified as a prevalent issue in sexually mature Muscovy ducks when housed in groups and provided with unrestricted access to food [62,63]. In the hybrids used for the production of foie gras, feather pecking and cannibalism appear to be less prevalent, both prior and during the force-feeding period [7,26]. In addition, when provided with access to open water, mulards have been observed to engage in bathing or swimming [60]. Ducks spend a considerable amount of time in displaying complex behaviour, which involves smoothing of their feathers [22]. When bathing after a meal, ducks perform several complex shaking motions to shake off the water. These cleaning movements are used to remove foreign objects and to spread fat from the uropygial gland, also known as the breech gland, which is located above the tail. This is necessary to make the feathers waterproof and to facilitate effective heat regulation. As waterfowl, ducks require access to both outdoor areas and water for bathing in order to meet their biological needs. If the animals are unable to bathe, they should be provided with sufficient water supplies designed so that ducks can immerse their heads in them and take up water with their beaks so they can effortlessly shake the water over their body [45]. Water provisions should be sufficiently accessible to avoid competition and should allow ducks to immerse the head and perform water-related preening behaviours. However, the design must also limit excessive spillage and deterioration of floor or litter quality. The optimal dimensions and configuration of such water facilities remain insufficiently established [26,45,64].

#### 4.3.2. Fear and Avoidance Behaviour

Ducks and geese that are hand-fed typically exhibit a positive response to the individual responsible for their feeding. Consequently, SCAHAW [7] visited farms practising force-feeding, yet no positive response towards the person executing the force-feeding was observed in these farms. It was observed that ducks exhibited a tendency to evade the force-feeder when the latter approached them, even though this evasion behaviour was associated with increased physical exertion by the ducks towards the end of the force-feeding period. However, when housed in individual cages, ducks appear to exhibit a preference for the caretaker who performs the force-feeding, as opposed to an unknown individual. Nevertheless, it can be concluded that the behaviour of the ducks during the force-feeding period does not reach optimal levels as ducks become stressed by the presence of unfamiliar individuals [43].

#### 4.3.3. The Aversion to the Feeding Probe

The welfare of birds may be compromised if they exhibit aversion to the force-feeding probe or to the caretaker who administers it. For instance, if the bird moves away when the caretaker attempts to catch it or if the bird pulls away from its head when the caretaker wants to insert the force-feeding tube, the feeding process takes longer. Furthermore, the non-cooperative behaviour of the bird may result in it being subjected to rough handling by the caretaker. The experimental evidence demonstrated that the birds that attempted to evade the caretaker or force-feeding tube were more likely to sustain injuries during the force-feeding process. Birds that demonstrate an ability to suppress such a reaction are less likely to exhibit signs of pain and other characteristics indicative of reduced well-being, in comparison to those that exhibit a strong reaction [26]. As previously stated, there are certain behavioural alterations that occur during the force-feeding period (see Section 4.3.1). The extent to which these behavioural changes may be indicative of aversion remains to be clarified. However, Rodenburg et al. [22] stated that headshaking serves as an indicator of aversion, which has also been observed in instances where ducks are denied access to open water [65]. Furthermore, the practice of force-feeding prevents the animals from displaying normal behaviour, such as species-specific foraging. It can thus be concluded that the animals are unable to satisfy their need to seek and consume food in order to achieve a state of satiety and homeostasis. This may result in a range of undesirable consequences, including frustration, a decline in welfare, and the manifestation of aversive behaviours [26].

#### 4.3.4. Thermoregulatory Behaviour

**Figure 1:**
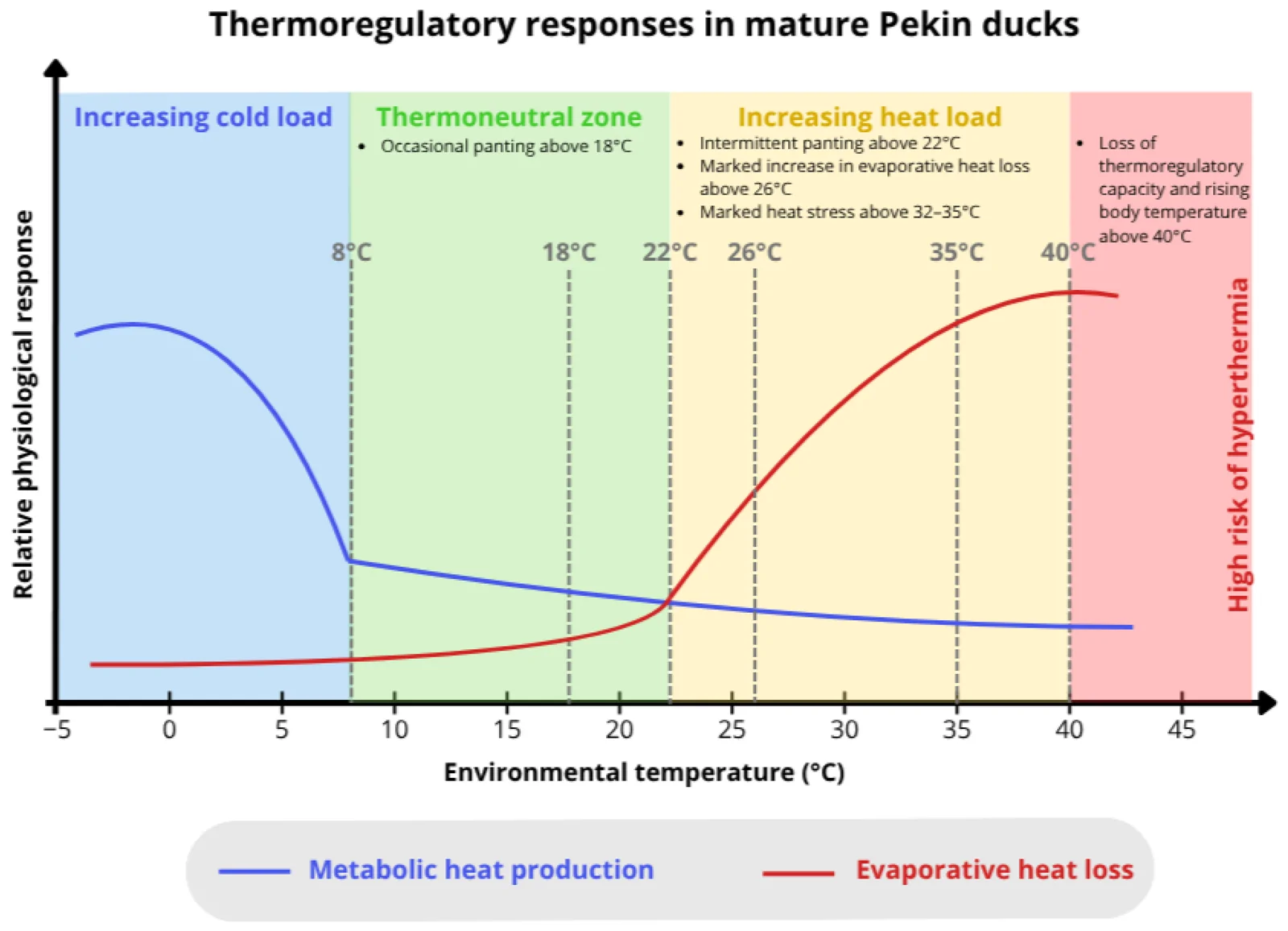
Conceptual representation of thermoregulatory responses in mature, fully feathered, ad libitum-fed Pekin ducks of approximately 3.6 kg. In the schematic relationship reported by Cherry and Morris [28], metabolic heat production is minimal within an approximate thermoneutral zone of 8–22 °C. Individual birds may occasionally pant above approximately 18 °C, while intermittent panting becomes evident above approximately 22 °C. Above approximately 26 °C, evaporative heat loss increases markedly, particularly at relative humidity above 60%. Behavioural signs of marked heat stress occur above approximately 32–35 °C. Above approximately 40 °C under relatively dry conditions, mature acclimatised ducks may no longer dissipate sufficient metabolic heat, causing body temperature to rise and creating a high risk of hyperthermia. Curves are schematic and thresholds approximate. Figure developed by the authors based on physiological principles and observations reported by Cherry and Morris [28].

The thermoneutral zone is the range of environmental temperatures within which metabolic heat production is minimal and body temperature can be maintained without additional energy expenditure for heating or active evaporative cooling. In the schematic relationship reported by Cherry and Morris [28] for mature, fully feathered, ad libitum-fed Pekin ducks of approximately 3.6 kg, this zone extends approximately from 8 to 22 °C (Figure 1). These boundaries are approximate and may shift with genotype, age, feed intake, physical activity, flooring, air speed, humidity and other environmental conditions. Normal body temperature in ducks is approximately 41 °C [28,66].

Heat dissipation can be achieved through a range of physiological and behavioural thermoregulatory mechanisms [66]. In cool conditions, birds primarily lose heat through non-evaporative (also called sensitive) mechanisms such as convection, conduction, and radiation. The magnitude of heat dissipation through non-evaporative pathways is contingent on the temperature gradient between the environment and the body surface, as well as the air speed. As the discrepancy in size diminishes and the airspeed decreases, the quantity of heat that can be released declines accordingly [28,66]. As the ambient temperature increases, the non-evaporative heat transfer process is reduced, and eventually ceases when the ambient temperature equals the duck’s body temperature [28]. Evaporative (or latent) heat losses are primarily attributable to the evaporation of moisture along the respiratory tract. Evaporation of moisture from the animal’s body is also a possibility, but for this to occur, the animal must have access to an external water source, as it is not naturally equipped with sweat glands [26,66]. The extent of evaporative heat loss is determined by environmental temperature and relative humidity. In the state of thermal equilibrium, evaporative heat losses account for approximately 20% of the total heat dissipation. In optimal conditions, this can be as high as 80% at 40% humidity; however, such low humidity is an uncommon occurrence in a stable environment [66].

Individual mature, fully feathered ducks may occasionally pant at ambient temperatures above approximately 18 °C, while intermittent panting becomes evident above approximately 22 °C. Above approximately 26 °C, particularly when relative humidity exceeds 60%, ducks substantially increase their respiratory rate to enhance evaporative heat loss, and some birds may use gular flutter. During panting, rapid, shallow breathing with an open bill increases evaporation from the upper respiratory tract. Gular flutter further promotes evaporative and forced-convective heat loss while limiting excessive alveolar ventilation and associated bicarbonate loss [28,66]. Above approximately 40 °C under relatively dry conditions, mature acclimatised ducks may no longer dissipate sufficient metabolic heat, causing deep body temperature to rise and creating a high risk of hyperthermia [28].

In ducks, panting is widely regarded as an indicator of discomfort. However, panting is a thermoregulatory reflex in birds, as it is in dogs. This is due to the absence of sweat glands [34,67]. Cherry and Morris [28] state that ducks exhibit a high level of tolerance for warm conditions (Table 1). This is partly attributable to the anatomical characteristics of ducks, namely their relatively small oesophagus and absence of a crop, which limits their capacity for feed storage. As a result, the metabolic heat production would be reduced, thereby minimising the animals’ experience of heat dissipation at lower ambient temperatures. However, in the context of force-feeding and the preparatory procedures that precede it, the oesophagus is stretched, and ducks are provided with large portions of feed. Force-fed ducks, or ducks on a high feed level, exhibit a reduced capacity to tolerate warm conditions in comparison to ducks on a normal diet. They may already be experiencing difficulties in terms of heat dissipation at lower ambient temperatures [26,46]. In the case of force-fed ducks, this overheating is partly due to the enlarged liver. In humans, the liver is responsible for 30% of the body’s total heat production. Therefore, a liver which has been enlarged eight-to tenfold will produce a greater quantity of heat [66]. Souvestre et al. [66] observed marked thermoregulatory changes between the beginning and end of the force-feeding period. Mean cloacal temperature increased from approximately 40.6 °C on day 1 (initiation of force-feeding) to 42.1 °C on day 11, while respiratory rate increased from 13 to 65 breaths per minute. Surface temperatures of the bill and legs also increased, which is consistent with increased peripheral heat dissipation [66]. The increase in cloacal temperature represents a substantial rise relative to the relatively narrow normal range of avian body temperature [28,66]. Because no infectious or pyrogenic cause was demonstrated, this increase should not be described as fever. The markedly increased respiratory rate, accompanied by reported panting, is more appropriately interpreted as evidence of increased metabolic heat load and a reduced capacity to maintain thermal equilibrium towards the end of the force-feeding period [66].

The information compiled, paraphrased and restructured in Table 1 by the authors comes from observations reported by Cherry and Morris [28]. A dash indicates that no additional specific observation was reported at that threshold. Temperature thresholds are approximate and apply to fully feathered ducks; responses may vary with age, genotype, feed intake, relative humidity, air speed, flooring and other environmental conditions.

The following management options have been proposed as potential interventions to support thermoregulation in force-fed ducks [66]. However, the reviewed evidence does not demonstrate that these measures are uniformly implemented in commercial production or that each intervention produces a consistent welfare benefit under commercial conditions. For example, monitoring and regulating the ambient temperature and airspeed, dehumidifying the air by removing the manure more frequently or by placing a dehumidifier, adding bicarbonate to the feed (unless the animal is in metabolic alkalosis), adjusting the feed portion or allowing the animals to cool their legs in the water. Infrared technology can also be used to monitor litter quality, locate cold or hot areas, and assess the thermal comfort of the birds [66].

#### 4.3.5. Social Behaviour

A range of behavioural characteristics have been identified in relation to mulards. On the one hand, they show signs of anxiety and fear towards humans, or ‘they are sensitive towards the environment’, as Laborde and Voisin [44] express. In the proximity of other individuals, these birds exhibit signs of distress, attempting to either escape or retreat to the rear of the cage. On the other hand, they exhibit a strong propensity for group participation and a high degree of social orientation towards their peers. Compared with both parental lines, mulards are more prone to social stress, show greater fear responses towards humans, and exhibit stronger panic reactions, which have been attributed to heterosis effects [26,67,68,69]. Their behaviour towards other ducks indicates that group housing is probably a beneficial environment for them [26,69]. However, their behaviour towards humans renders mulards unsuitable for force-feeding, as these ducks display fear and nervousness in contact with humans. Caretakers engaged in force-feeding claim that, in addition to genetics, the provenance of the birds is a determining factor in the manifestation of their temperament as either calm or nervous [44]. Laborde and Voisin [44] investigated which management practices and experiences prior to the force-feeding period could influence the behaviour of the birds during force-feeding. It was discovered that different aspects of husbandry and management of ducks appeared to influence their behaviour while force-feeding, but the differences observed were not very pronounced. Ducks that were handled more frequently, for instance for weighing or vaccinations, and had more contact with humans, for example by manual feeding instead of automated feeding, exhibited a reduced level of nervousness. Laborde and Voisin [44] stated that there was a correlation between abrupt transfers from the previous site to the new site and elevated levels of nervousness in later force-feeding procedures.

In group housing systems, ducks have opportunities for social interaction and greater freedom of movement than in individual cages. However, the usable floor area per bird cannot be compared reliably across studies because group size, stocking density and system design are not consistently reported [26,67,70]. In comparison with birds housed individually, those in group housing systems have greater opportunity to rotate, extend their wings fully, and elevate to their maximum vertical height. This is a positive development with regard to animal welfare. Other factors influencing the welfare of animals in group housing include group size, stocking density, social interactions, floor type, light scheme and intensity, presence and quality of litter, access to an outdoor run, access to drinking water and the presence of water for bathing or at least submerging the head. In group housing systems, the control of air quality and ventilation, the cleanliness of the stable and birds, disease control and the maintenance of homogeneous groups with regard to body weight must be given due consideration [26].

Whilst group housing offers numerous advantages for the birds, it is imperative to consider potential adverse effects. Competition for water resources has been identified as a potential cause of aggression among the birds. Furthermore, the apprehension of the birds for the purpose of the force-feeding procedure has the potential to induce heightened levels of stress and anxiety. Maintaining hygiene, particularly in larger groups, may present a greater challenge as well [26]. Recent studies have indicated that housing conditions during the force-feeding period significantly influence behavioural responses and welfare outcomes. Ducks housed in floor pens exhibit more natural behaviours and show lower levels of agitation and struggling during feeding compared to ducks kept in elevated pen systems [61].

On balance, group housing permits social contact and greater behavioural freedom than individual cages and avoids the severe spatial and behavioural restrictions inherent to individual confinement [26,45,67,70]. However, poorly designed or managed group systems may increase disturbance, competition for resources, collisions, trampling or hygiene-related risks [26,61,67,70]. Group housing should therefore be regarded as preferable in principle, but acceptable welfare remains dependent on usable floor area, flooring, group size and composition, stocking density, access to water and other resources, environmental conditions and the condition of individual birds [23,26,45,61,67,70].

In general, domesticated animals establish a certain relationship with their caretakers, particularly when the caretaker provides positive resources, such as feed and litter, and engages in activities, such as talking to and caring for the animals. Broom and Rochlitz [26] interpreted resistance and attempts to escape during capture and immobilisation as possible evidence that ducks may find components of handling or force-feeding aversive. However, these responses do not allow aversion to the caretaker, the administered diet and the complete procedure to be distinguished from one another. Resistance and attempts to escape are compatible with a negative or aversive experience, but they are not specific indicators of pain and should be interpreted together with the circumstances in which they occur and with other welfare indicators [26,71].

### 4.4. Hygiene

The condition or hygiene of the nostrils, eyes and feathers of ducks is indicative of their ability to completely submerge their heads in the water and maintain their faces clean [26]. Feather smoothing and wing stretching are behaviours that are associated with a good welfare status in birds [22].

### 4.5. Lameness

The process of force-feeding results in the dilatation of the liver, consequently leading to the expansion of the abdomen. In order to maintain their balance, the birds will keep their legs further away from the midline of their body. However, this position complicates locomotion [7]. The use of small cages, which do not permit the birds to stand upright, hinders the discernment of leg problems and discomfort [26].

SCAHAW [7] and Rochlitz and Broom [26] suggested that an abnormal mineral balance in the diet of force-fed ducks might contribute to bone pathology, with the mechanical consequences potentially being exacerbated by increased body mass. However, direct duck-specific evidence demonstrating that nutritional imbalance causes clinically relevant bone pathology during the relatively short force-feeding period is limited. The short duration of this period and the availability of body mineral reserves may limit the development of nutritionally induced bone pathology, but this has not been established directly in force-fed ducks [7,26].

Overweight reduces mobility due to increased pressure on the legs, reduced breathing capacity and pain. In the event of decreased movement by the birds, the bones and muscles will not develop as well as they would under normal activity conditions. The risk of skin damage also increases and social interactions with peers change [26].

Bone fractures reported in force-fed ducks have predominantly involved the wing bones, particularly the humerus [7,72]. Bénard [72] reported a fracture prevalence of 30–70% in mulards following handling before slaughter, compared with approximately 5% in Muscovy ducks. The frequently cited prevalence of 30–70% is derived from a study published in 1992 and may not represent current genotypes, housing, equipment, catching or handling practices [72]. No recent study providing directly comparable prevalence data was identified in this review, which constitutes an important evidence gap. The observed variation in the prevalence of fractures appeared to be associated with the quality of care provided by the staff and the prevailing climatic conditions. The birds exhibited elevated levels of nervousness in adverse weather conditions, which resulted in an increased incidence of fractures [7,72]. Furthermore, other factors have been identified as contributing to an increased risk of fractures and stress during transport, including fear of humans and the significantly higher thermoregulation requirements compared to normal ducks [26].

### 4.6. Injuries

Short-term changes in plumage or superficial feather condition do not necessarily demonstrate pain or stress. Nevertheless, poor plumage condition may indicate that the environment restricts bathing, preening or other body-maintenance behaviours. Where changes are accompanied by skin lesions, inflammation or pressure wounds, they may directly impair welfare through pain and discomfort [7,22,26]. A percentage of ducks that are force-fed and kept in individual cages show pressure wounds on the sternum and are more susceptible to broken bones during transport and slaughter [7,72]. The presence of injuries on the head provides information on the physical damage caused by the force-feeding procedure [26]. Sternum injuries can be both extensive and prevalent, particularly towards the end of the force-feeding period, with a higher incidence observed during the winter months. Loss of feathering in the chest area is also noted, mostly during the growth phases. It has been observed that the process of slaughtering can result in damage to the wings. Approximately 90% of the wing injuries are recent, and most likely occurred during the capture of the animals, their transportation to the slaughterhouse, and the fixation of animals prior to slaughter. Post-slaughter observations have revealed the presence of additional physical injuries, including scratches on the dorsal part of the body, pseudo-crop lesions, and joint abnormalities. In general, the prevalence of injuries varies greatly between different farms and groups of birds. The variation cannot be explained by parameters such as stocking density and season [26]. Several of these lesions are not unique to force-fed ducks. Footpad dermatitis, breast lesions, scratches and wing injuries may also occur in ducks reared for conventional meat production and are influenced by flooring, litter moisture, stocking density, catching, handling and transport. Their occurrence in conventional meat ducks does not exclude a contribution from force-feeding-related conditions. Increased body mass, reduced activity and the flooring, space allowance and handling associated with force-feeding housing may exacerbate the frequency or severity of some lesions [7,26,70]. In contrast, lesions of the oesophagus or pseudo-crop are more directly associated with repeated tube insertion during force-feeding. Robust comparisons remain limited because few studies have compared force-fed and non-force-fed mule ducks under otherwise identical housing and management conditions [7,26,43,59].

Pododermatitis, also known as footpad dermatitis or contact dermatitis, is a painful condition in which inflammation and necrotic lesions are present on the plantar surface of the soles of feet and toes. The severity of the lesions can range from superficial to deep infestations. These deep ulcers have the potential to evolve into abscesses and to cause thickening of the underlying tissues and structures [73,74]. Such dermatological conditions, including hock burns and breast blisters, are not typically of bacterial origin. Instead, they are caused by unsuitable flooring.

In mulards, Mirabito et al. [70] compared several production and welfare-related parameters between individually housed ducks and ducks housed in group cages. The parameters included final body weight, magret, liver and thigh weight, and the prevalence of carcass lesions. The prevalence of injuries and scratches on the back were more common in ducks in group housing than in individually housed ducks. This observation aligns with the hypothesis that aggression among birds may be a contributing factor. Conversely, in the case of humeral head injuries, a contrary phenomenon was observed. A greater proportion of such injuries were identified in individually housed ducks during post-mortem examination, yet disparities were also discerned within group cages. Ducks housed at the highest stocking density in group cages had more humeral lesions at the time of slaughter in comparison to ducks housed at the lower stocking density. This phenomenon may be attributed to the reduced activity levels and be explained by the reduced activity and consequently weaker bones observed in ducks housed at relatively high stocking densities [26,70].

There is a higher prevalence of severe breast injuries in birds housed on plastic slatted floors compared to steel slatted floors [26,70]. Furthermore, the type of litter, or the quality of the ground or surface, in the absence of litter, is an important contributing factor, particularly in the initial stages of contact dermatitis. Damage occurs on skin surfaces that are in prolonged contact with the litter. Initially, this is observed on the soles and toes, followed by the back of the heels and, in severe cases, the chest. Although excess moisture is sufficient to cause the condition, there are other causative factors. A number of factors have been identified as influential in this area, including litter thickness and quality, ammonia content, climatic conditions such as condensation and ventilation, stocking density, the rearing system, leg weakness and the diet [74,75,76,77].

### 4.7. Metabolism

Mulards are considered to be a suitable species for foie gras production, due to their anatomical characteristics that facilitate the accumulation of fat, predominantly in the liver, with less accumulation occurring in other parts of the body [78,79].

Metabolic diseases frequently manifest with minimal or non-apparent clinical indications. Consequently, these conditions cannot be readily diagnosed in the absence of abnormal physiological changes that can be observed through the implementation of an appropriate clinical biochemical method. Furthermore, these abnormalities do not necessarily imply suffering. In addition, there are functional diseases which occur in the absence of clearly demonstrable biochemical abnormalities, but which are associated with painful symptoms. The distinction between a functional disease and the pre-clinical stage, or slowly progressive disease, is not always possible, especially in animals whose lifespan is limited by the farming system. The manifestation of lesions within innervated anatomical regions is accompanied by the experience of discomfort. In other anatomical regions, these phenomena can result in deformities that appear atypical but are not necessarily associated with discomfort [7]. The rapid introduction of a large quantity of feed through the use of force-feeding has been demonstrated to increase heat production, which in turn has been shown to induce panting and the production of semi-liquid manure.

In addition to the aforementioned points, it is important to acknowledge that an enlarged liver can exert pressure on the air sacs, thus diminishing the respiratory capacity and potentially compressing other abdominal organs. In cases where liver function is severely compromised, the onset of hepatic encephalopathy is a potential outcome [7,80].

### 4.8. Growth and Body Weight

Growth and body-weight trajectories are relevant to the assessment of force-fed ducks only insofar as abrupt or excessive changes are associated with animal-based or functionally relevant outcomes. Rapid increases in body and liver mass may contribute to locomotor burden, reduced activity, altered resting behaviour and increased metabolic heat production [7,26,61,66]. Conversely, failure to maintain the expected feed intake or body-weight trajectory may indicate impaired health or an inability to cope with the feeding programme. Body weight for age is therefore a contextual production indicator and health measure rather than a stand-alone welfare indicator and should be interpreted together with behaviour, mobility, thermoregulatory responses, morbidity and mortality [7,26].

### 4.9. Mortality

Historical data reported by SCAHAW [7] indicated mortality rates of approximately 2–4% during a two-week force-feeding period, compared with approximately 0.2% over a comparable period in ducks of the same age reared for meat without force-feeding. This represents a roughly 10–20-fold numerical increase. However, the groups also differed in housing, relocation, diet and management, and the underlying causes of death were not consistently established [7,26]. The comparison therefore suggests a potentially important welfare concern but does not isolate force-feeding as the sole cause of the observed difference. Robust contemporary comparisons would require force-fed and non-force-fed mulards maintained under otherwise comparable housing, environmental and management conditions.

The potential consequences of liver failure may include hepatic necrosis, haemorrhages of the liver and blood poisoning. Respiratory problems, intestinal disorders and cardiomyopathy can also result in death. As the length of the force-feeding period was found to be positively correlated with mortality, it is recommended that this period is kept as short as possible [8,19].

SCAHAW [7] mentions additional mortality figures; however, these data are outdated and pertain to ducks housed individually during the force-feeding period. SCAHAW [7] reported the findings of a veterinary inspector in Belgium, who observed an average mortality rate of 2.75%, based on data from 77,519 ducks at 16 farms. However, the mortality rates exhibited substantial variation between farms and batches, ranging from 0 to 15%. There was a higher mortality rate during the summer months than during the other seasons. This seasonal pattern is consistent with an increased thermoregulatory risk, but the observational data do not establish heat load as the sole cause of mortality [7]. Nevertheless, the finding supports particular attention to ambient temperature, relative humidity, ventilation, air movement and the timing of production, especially towards the end of the force-feeding period when cloacal temperature and respiratory rate may be elevated [7,26,66]. Estevez (s.a., in SCAHAW [7]) mentioned a mortality rate of a Spanish farm that raised between 34,000 and 55,000 birds per year. The annual rate of mortality varied between 0.9% and 1.1% over a period of seven years.

### 4.10. Health

The health status of the birds can be evaluated by means of a number of parameters, including but not limited to body anatomy, posture, mobility and gait, face, body condition, the plumage, the presence of (severe) skin lesions (sole, toe and heel dermatitis, breast lesions) and mortality [81,82]. Force-feeding has been associated with time-dependent changes in the intestinal microbiota of mulard ducks, including changes in microbial diversity and the relative abundance of particular bacterial taxa [83]. Rapid dietary change, high starch intake and altered digesta flow may contribute to these changes, but their independent effects have not been established. Associations between microbiota composition, inflammatory or metabolic markers and force-feeding stage do not by themselves demonstrate that microbiota changes cause diarrhoea, intestinal lesions or impaired welfare. Targeted longitudinal and intervention studies are required to determine whether particular microbial changes contribute causally to gastrointestinal disease or adverse welfare outcomes.

## 5. Alternatives to Force-Feeding

Despite the Standing Committee of the Council of Europe’s [45] recommendation to encourage research into alternatives to force-feeding of ducks for the production of foie gras, scientific research has historically concentrated on force-feeding-related aspects, including stress physiology, feed composition, slaughter aspects and (epi)genetic effects and not on the development of alternative feeding techniques to replace force-feeding-based foie gras production [43,67,84,85,86]. Despite the investment in research over several decades, the evaluation of welfare during the force-feeding period remains a complex aspect. This complexity is partly attributable to the lack of validated behavioural indicators and methodological inconsistencies between studies [61].

In addition to the paucity of research regarding alternatives, the majority of studies focus on geese rather than ducks. Existing research is frequently exploratory in nature, primarily proposing potential directions for exploration rather than providing substantiated alternative production methodologies. In addition to feeding strategies, housing conditions (e.g., group size, floor type) and management practices (e.g., feeding schedules, lighting regimes) are likely to play an important role. Furthermore, it is generally anticipated that alternative methods will yield a reduced degree of hepatic steatosis [4]. Consequently, the question arises as to whether such a product is perceived and accepted by consumers as foie gras.

Guy et al. [87] observed that the occurrence of fatty liver disease in geese does not necessarily require the utilisation of force-feeding techniques. Following a period of feed restriction, the geese were provided with a maize-based diet, and the lighting was limited to between 7 and 10 h per day. This can be regarded as a light simulation of autumn, during which geese accumulate fat reserves. However, the researchers noted that the variation in absolute and proportional liver weight is substantial and highlighted the difficulties of standardising such approaches under commercial conditions.

Information pertaining to spontaneous liver fattening in geese has also been reported in non-scientific sources, including websites and forums. This production model encompasses the production of ’Pateria de Sousa’ in Spain and the Aviwell project (www.aviwell.fr) in France. The findings of both studies suggest that the accumulation of liver fat in geese is possible without the use of force-feeding techniques. However, the available information on these production processes remains limited, and the practical, qualitative and financial feasibility of the production is also unclear.

Reports concerning the Aviwell initiative indicate that findings from human obesity and microbiome research were used as a hypothesis-generating basis for exploring whether probiotic or microbiota-directed interventions might promote hepatic lipid accumulation in geese without force-feeding [88]. Evidence from human microbiome research does not demonstrate that the same mechanisms will induce hepatic steatosis safely, predictably or reproducibly in geese. Moreover, the available information is largely commercial and non-peer-reviewed, and controlled studies evaluating liver function, animal health, welfare, product consistency and longer-term outcomes were not identified. Microbiome-based interventions should therefore currently be regarded as a commercially promoted but scientifically unvalidated research direction rather than as an established alternative production method. Advances in cellular agriculture have also led to increased interest in cultured or cell-based foie gras products. These technologies are frequently presented as potential alternatives to conventional animal production systems [89]. In addition to plant-based spreads, recent biotechnological advancements have led to the development of cultivated duck liver cells. This breakthrough allows the production of cell-based foie gras without the necessity of force-feeding the ducks [90]. Several companies are currently exploring such approaches, including initiatives in Europe, the United States, and Asia. However, the majority of these initiatives are still in the early stages of development, and there is a paucity of peer-reviewed data on their feasibility, safety, and product quality.

Recent research has explored technological approaches to recreate foie gras-like products without force-feeding. These approaches focus on modifying the physicochemical properties of liver–fat emulsions, particularly the structure and distribution of fat droplets, which are key determinants of texture and mouthfeel [91]. A range of commercial products is marketed using terms such as “fatty goose liver”, “ethical foie gras”, “humane foie gras” or “foie gras alternative” [92,93,94]. These terms do not constitute standardised production or animal-welfare classifications and may refer to substantially different products and production methods. Marketing claims such as “ethical” or “humane” should therefore specify whether force-feeding is entirely absent and should be supported by transparent information on production methods and relevant welfare outcomes. Systems that retain force-feeding but modify tube material, housing, handling or other procedural conditions should be described as modified conventional systems rather than as force-feeding- free alternatives.

Other products avoid force-feeding entirely but differ in how closely they resemble conventional foie gras. Foie Royale is reported to be produced by combining liver from ducks or geese reared for meat with species-specific fat and processing the mixture under high pressure to obtain a texture resembling conventional foie gras [93]. This approach uses animal-derived ingredients but does not require the production of a greatly enlarged fatty liver through force-feeding. Faux Gras de GAIA is a plant-based spread intended to approximate some of the sensory characteristics and culinary uses of foie gras [94]. Cultivated-cell products constitute a further category in which duck cells are used to produce foie gras-like tissue without force-feeding birds [90]. These categories should be evaluated separately because they differ in animal use, production method, product composition, technological maturity and potential welfare implications.

Several approaches have been proposed to avoid force-feeding, including spontaneous or voluntary hyperphagia-induced fattening, nutritional or microbiota-directed interventions, cultivated-cell production and plant-based or processed liver–fat analogues [87,88,89,90,91,92,93,94]. The evidence remains limited and fragmented. This may reflect technical difficulties, seasonal biological constraints, product-quality requirements, intellectual-property and commercial confidentiality, uncertain investment and the position of conventional foie gras within an established cultural and gastronomic market. However, the relative contribution of these factors has not been quantified. No animal-based alternative has yet been shown in peer-reviewed research to produce fatty livers with consistent composition and technological properties at commercial scale while demonstrating that animal welfare is not compromised and, where possible, is improved relative to force-feeding.

Consumer acceptance is likely to influence practical feasibility [1]. Alternatives should therefore be evaluated for safety, sensory properties, price and perceived cultural or culinary authenticity. Fernandez et al. [39] demonstrated that sensory differences can occur, but consumer acceptance and willingness to pay for such alternatives have not been established. These criteria should nevertheless be assessed separately: similarity in taste or texture does not demonstrate improved animal welfare, while improved welfare does not by itself establish product safety, commercial viability or consumer acceptance. Future evaluations should therefore report production method, animal use, force-feeding status, welfare outcomes, product composition, safety, sensory quality, scalability and cost transparently.

## 6. Welfare Assessment and Improvement

### 6.1. Translating Current Evidence into Welfare Improvement

A multidisciplinary framework should combine animal welfare science, behaviour, physiology, pathology, veterinary medicine and nutrition [7,23,26,46]. Agricultural engineering and precision livestock farming (PLF) should be included for housing and environmental control and for the development of automated monitoring, together with economic analysis of implementation and the feasibility of alternative systems. Behavioural, clinical, physiological, pathological and health-related outcomes do not necessarily change in parallel; consequently, an improvement in one outcome should not automatically be interpreted as an overall improvement in welfare [7,26,46,51,61].

Monitoring should prioritise animal-based indicators that can reveal meaningful changes during the force-feeding period. Camera-based or other automated monitoring may support the assessment of gait and mobility, posture, activity, resting, preening, avoidance behaviour, respiratory rate and persistent panting. Direct inspection remains necessary for injuries to the bill or upper digestive tract, footpad and breast dermatitis, body and plumage condition and gastrointestinal signs such as regurgitation, diarrhoea or abnormal droppings, as well as morbidity and mortality [7,26,42,61,66,70,81]. Interpretation depends on housing, production stage, body and liver mass, environmental conditions, including temperature and relative humidity, and previous experience with humans [7,26,43,44,61]. Indicators should therefore be interpreted together rather than in isolation.

### 6.2. Monitoring and Intervention Thresholds

Monitoring should combine individual-animal observations with flock-level records and information on feeding procedures, housing and environmental conditions. Flock-level averages may conceal severely affected individuals, particularly when lesions, mobility or mortality vary among farms and batches [7,26]. Birds showing marked locomotor impairment, persistent panting, abnormal posture, injuries, severe dermatitis, gastrointestinal disturbances or other clinical abnormalities warrant individual assessment. Mortality should be recorded according to the stage of force-feeding and, where possible, investigated through veterinary post-mortem examination to identify lesions or diseases not apparent during routine observation [7,26,70].

Validated thresholds specifying when force-feeding should be modified, temporarily suspended or discontinued for an individual bird have not been established in the literature reviewed [7,26,61,66,70]. Threshold-development studies should be prospective and adequately powered, include repeated measurements before and throughout force-feeding to establish individual trajectories rather than relying solely on flock means, and represent relevant genotypes, housing systems, management practices and climatic conditions. Candidate thresholds should be linked prospectively to subsequent deterioration or recovery and tested for validity, repeatability, sensitivity, specificity and feasibility under commercial conditions. Combinations of indicators may prove more informative than single measures, but their added predictive value must be demonstrated rather than assumed.

Technological monitoring may complement, but should not replace, direct observation by farmers and clinical examination by veterinarians. Infrared thermography and other non-invasive temperature measurements may help detect changes in heat dissipation, particularly towards the end of the force-feeding period [66]. Their use for welfare-related decision-making requires validation against relevant behavioural, physiological, pathological and clinical outcomes.

### 6.3. Procedural, Housing, and Environmental Management

Operator competence and equipment condition remain relevant because inappropriate restraint or tube insertion may increase the risk of injury to the bill, oral cavity, pharynx and oesophagus [7,26,42]. Familiarity with the operator and previous human contact may also influence avoidance and nervousness [43,44]. These findings support consistent and careful handling by trained personnel using properly maintained equipment. However, the independent welfare effects of tube design and dimensions, insertion speed, feed quantity, administration rate, feeding frequency and degree of automation have not been quantified sufficiently to identify specific procedural modifications as validated welfare improvements [7,26,38,40,42,46].

Housing design should provide sufficient usable space for simultaneous resting, postural changes and social contact while limiting competition, collisions, trampling and hygiene problems. Group size, stocking density, flooring, litter condition and access to water may affect activity, body-maintenance behaviour and the prevalence of breast, footpad, carcass and humeral lesions [22,26,45,61,64,67,70]. Modifications should be evaluated across several welfare domains because an intervention may improve one outcome while adversely affecting another. Evidence obtained from ducks outside the force-feeding period should not automatically be extrapolated to force-fed birds, particularly towards the end of the production period when mobility and motivation to use resources may be altered.

Environmental control requires particular attention because force-fed ducks may have an increased metabolic heat load and a reduced capacity to maintain thermal equilibrium. Ambient temperature, relative humidity, ventilation and air movement should be recorded together with panting, respiratory rate, posture and, where feasible, body or surface temperature measurements [7,26,66]. The higher summer mortality reported in historical data is consistent with thermoregulatory risk but does not establish heat load as the sole cause [7]. Proposed environmental interventions should therefore be evaluated using animal-based outcomes under commercial conditions rather than assumed to produce a uniform welfare benefit.

### 6.4. Research Priorities and Implementation

Future studies should use longitudinal or controlled designs that distinguish the effects of handling and tube insertion from those associated with feed quantity, progressive body and liver enlargement, housing and environmental conditions. Reports should describe genotype, age, housing, usable floor area, group size, flooring, force-feeding protocol, equipment, operator experience, environmental conditions and timing of measurements in sufficient detail to allow comparison and replication [7,26,51,61]. Studies should assess behavioural responses, mobility, injuries, physiological and metabolic changes, thermoregulation, morbidity and mortality alongside production outcomes.

Research on implementation should consider technical effectiveness, welfare effects, operator requirements and economic feasibility together. For force-feeding-free alternatives, assessment should additionally include scalability, product safety and consistency, sensory properties, consumer acceptance, price and cultural context [1,39,87,88,89,90,91,92,93,94]. Production performance or product similarity should not substitute for animal-based welfare evidence, while a potential welfare improvement does not by itself establish commercial viability. Transparent reporting across these domains is required to identify interventions and alternatives that are both effective and practically applicable.

## 7. Conclusions

The available evidence indicates that force-feeding creates substantial risks of welfare compromise through repeated restraint and tube insertion, possible injuries to the upper digestive tract, progressive liver enlargement, altered hepatic function, increased metabolic heat load and impaired mobility. Historical data also indicate elevated mortality during the force-feeding period relative to non-force-fed ducks of comparable age, although interpretation is complicated by differences in housing and management. Endocrine findings are equivocal, and plasma corticosterone concentrations alone cannot establish either the presence or absence of pain, fear, distress or other adverse welfare states. Welfare assessment should therefore integrate behavioural, clinical, physiological, pathological and health-related outcomes rather than rely on any single indicator. Housing, operator competence and environmental management may modify particular risks but cannot eliminate repeated exposure to restraint, tube insertion and the forced administration of large quantities of feed. Evidence concerning force-feeding-free alternatives remains limited, particularly regarding their welfare benefits and commercial feasibility. Future research should use controlled longitudinal designs and a validated, integrated set of animal-based outcomes to distinguish the effects of force-feeding from those of housing, handling and environmental conditions, and to evaluate conventional, modified and alternative production systems transparently.

## Figures and Tables

**Table 1:** Reported behavioural and thermoregulatory responses of fully feathered ducks at different ambient temperatures.

Ambient Temperature	Posture and Resting Location	Activity, Feeding and Social Behaviour	Reported Thermoregulatory Response
≤5 °C	Group huddling, mainly on litter; legs tucked beneath the body; head may be placed beneath a wing.	Reduced feeding and drinking; unusually low general activity.	Grouping and tucked postures reduce exposed surface area and limit heat loss.
12 °C	Resting primarily on litter, with the legs remaining exposed.	Feather maintenance and apparently undisturbed resting behaviour.	No specific evaporative cooling response described.
≥15 °C	Resting on litter, with the legs remaining exposed.	General social activity increases; feather maintenance continues.	No specific evaporative cooling response described.
≥18 °C	Resting groups become smaller; greater use of wire or slatted flooring; legs and feet remain exposed.	Drinking, feather maintenance and social activity increase.	Occasional panting may occur in individual birds.
≥22 °C	-	-	Panting begins intermittently.
≥26 °C	-	-	Panting becomes very frequent, particularly when relative humidity exceeds 60%; some birds use gular flutter.
≥32 °C	Birds become increasingly lethargic and reluctant to move.	Little or no feeding or social activity; birds remain unusually quiet.	Behavioural signs indicate a substantial heat load.
≥35 °C	Birds sit with the head and neck extended.	Birds appear increasingly distressed.	Panting with gular flutter; water may drip from the bill.

## Data Availability

No new data were created or analysed in this study. Data sharing is not applicable to this article.

## Abbreviations

The following abbreviations are used in this manuscript:

ACTH	Adrenocorticotropic hormone
AVMA	American Veterinary Medical Association
BSP	Bromosulfophthalein
ChREBP	Carbohydrate Response Element-Binding Protein
CIFOG	Comité Interprofessionnel des Palmipèdes à Foie Gras
EC	European Commission
EFSA	European Food Safety Authority
EU	European Union
ICG	Indocyanine Green
INRA	Institut National de la Recherche Agronomique
SCAHAW	Scientific Committee on Animal Health and Animal Welfare
SREBP-1c	Sterol Regulatory Element-Binding Protein 1c
TED	Tracheal and Esophageal Displacement
VLDL	Very Low Density Lipoprotein
